# Identification of prognostic signatures in remnant gastric cancer through an interpretable risk model based on machine learning: a multicenter cohort study

**DOI:** 10.1186/s12885-024-12303-9

**Published:** 2024-04-30

**Authors:** Zhouwei Zhan, Bijuan Chen, Hui Cheng, Shaohua Xu, Chunping Huang, Sijing Zhou, Haiting Chen, Xuanping Lin, Ruyu Lin, Wanting Huang, Xiaohuan Ma, Yu Fu, Zhipeng Chen, Hanchen Zheng, Songchang Shi, Zengqing Guo, Lihui Zhang

**Affiliations:** 1 Department of Medical Oncology, Clinical Oncology School of Fujian Medical University, Fujian Cancer Hospital, No. 420 Fuma Road, Fuzhou, Fujian 350014 People’s Republic of China; 2 Department of Radiation Oncology, Clinical Oncology School of Fujian Medical University, Fujian Cancer Hospital, Fuzhou, Fujian 350014 People’s Republic of China; 3 Department of Pathology, Shengli Clinical Medical College of Fujian Medical University, Fujian Provincial Hospital, Fuzhou, Fujian 350001 People’s Republic of China; 4 Department of Hepatobiliary and Pancreatic Surgery, Clinical Oncology School of Fujian Medical University, Fujian Cancer Hospital, Fuzhou, Fujian 350014 People’s Republic of China; 5 Department of Pharmacy, Clinical Oncology School of Fujian Medical University, Fujian Cancer Hospital, Fuzhou, Fujian 350014 People’s Republic of China; 6 School of Basic Medical Sciences of Fujian Medical University, Fuzhou, Fujian 350004 People’s Republic of China; 7 Department of Critical Care Medicine, Shengli Clinical Medical College of Fujian Medical University, Fujian Provincial Hospital South Branch, Fujian Provincial Hospital, Fuzhou, Fujian 350001 People’s Republic of China

**Keywords:** Remnant gastric cancer, Prognosis, Prediction model, Machine learning, Interpretable, Multicenter

## Abstract

**Objective:**

The purpose of this study was to develop an individual survival prediction model based on multiple machine learning (ML) algorithms to predict survival probability for remnant gastric cancer (RGC).

**Methods:**

Clinicopathologic data of 286 patients with RGC undergoing operation (radical resection and palliative resection) from a multi-institution database were enrolled and analyzed retrospectively. These individuals were split into training (80%) and test cohort (20%) by using random allocation. Nine commonly used ML methods were employed to construct survival prediction models. Algorithm performance was estimated by analyzing accuracy, precision, recall, F1-score, area under the receiver operating characteristic curve (AUC), confusion matrices, five-fold cross-validation, decision curve analysis (DCA), and calibration curve. The best model was selected through appropriate verification and validation and was suitably explained by the SHapley Additive exPlanations (SHAP) approach.

**Results:**

Compared with the traditional methods, the RGC survival prediction models employing ML exhibited good performance. Except for the decision tree model, all other models performed well, with a mean ROC AUC above 0.7. The DCA findings suggest that the developed models have the potential to enhance clinical decision-making processes, thereby improving patient outcomes. The calibration curve reveals that all models except the decision tree model displayed commendable predictive performance. Through CatBoost-based modeling and SHAP analysis, the five-year survival probability is significantly influenced by several factors: the lymph node ratio (LNR), T stage, tumor size, resection margins, perineural invasion, and distant metastasis.

**Conclusions:**

This study established predictive models for survival probability at five years in RGC patients based on ML algorithms which showed high accuracy and applicative value.

**Supplementary Information:**

The online version contains supplementary material available at 10.1186/s12885-024-12303-9.

## Introduction

Remnant gastric cancer (RGC), also known as gastric stump cancer, was initially reported by Balfour in 1922 as a cancer developing in the remnant stomach following previous gastric surgery for peptic ulcer disease (PUD)[1, 2]. More recently, the definition of RGC has evolved, and it is now described as any cancer occurring in the residual stomach following a previous partial gastrectomy for benign or malignant conditions[3]. In literature, the incidence of RGC ranges approximately from 1 to 7%[4–8]. Due to the absence of specific symptoms, RGC is often diagnosed at an advanced stage, resulting in low surgical resection rates and poor prognoses, making it an important clinical concern[4, 5]. The surgical outcomes for RGC vary across studies, with 5-year survival rates ranging from 7 to 80%[6, 9–12].

As the number of gastrectomies continues to rise, the incidence of RGC is escalating annually[13]. It’s crucial to identify relevant prognostic factors for RGC and develop effective follow-up treatment strategies. In clinical practice, the adjacent gastric mucosa in RGC demonstrates a lower degree of atrophy when compared to cases of primary gastric cancer (GC), which suggests a unique underlying pathological mechanism[14]. Furthermore, there is a significantly heightened incidence of serosal tumor invasion in RGC, affecting between 37 to 48% of patients, contrasting sharply with the rate of 19% seen in primary GC[15]. Additionally, surgical procedures for RGC result in a notably smaller total number of harvested lymph nodes compared to those in primary GC, particularly when the preceding surgery was for gastric malignancy, since the nodes would have already been removed. As such, the lymph node grouping applied in the TNM classification system for primary GC may not be suitable for staging RGC[16]. Moreover, RGC shows a significantly higher overall frequency of splenic hilar lymph node involvement when compared to primary GC. It is worth noting that jejunal mesentery lymph node involvement is predominantly observed following Billroth II reconstruction surgeries[17, 18].

RGC often exhibits a higher rate of invasion into adjacent organs, and lymph node metastasis is frequently observed[19], which can lead to a worse prognosis than primary GC[20]. However, some studies suggest that RGC prognoses are similar to primary GC[21]. Prior research has investigated the clinical characteristics of resectable RGC in small case studies, but the factors influencing patient outcomes remain unclear or controversial[22–24]. A meta-analysis disclosed that the significance of tumor location on survival varies among studies. Some literature indicates that tumor location does not significantly impact survival rates[25, 26], while other research reports that anastomotic site tumors may be a favorable prognostic factor[27]. Nonetheless, patients with anastomotic site tumors experience worse outcomes[23]. Thus, additional research is necessary to resolve this discrepancy.

Machine learning (ML) constitutes the bedrock of contemporary artificial intelligence advancements[28]. Although these algorithms have demonstrated substantial triumphs across various disciplines, their integration into the realms of medicine and healthcare is still in its nascent stages. The non-linear nature of real-world data impacts often challenges the effectiveness of traditional models like Linear Regression for classification forecasts and Cox Regression for predicting survival outcomes, as they are confined within a linear framework[29, 30]. In comparison with traditional mathematical models, ML excels notably in handling tasks related to classification and regression, finding broad application in developing predictive frameworks, determining tumor stages, and prognostic groupings[31–34].

ML can facilitate various problems, from patient-level observations to employing algorithms with numerous variables, seeking combinations, and ultimately reliably predicting risks and outcomes[35]. Numerous studies have developed valuable models utilizing ML techniques[36–39]. However, there is a dearth of research exploring the application of ML for predicting survival outcomes in RGC patients. Although ML presents significant benefits in constructing models to identify risk factors, the “black-box” nature of ML algorithms poses challenges in explaining why specific predictions are made for patients. In pursuit of these objectives, the SHapley Additive exPlanations (SHAP) methodology has recently been introduced[40, 41]. The SHAP method allows for the recognition and prioritization of attributes that influence complex classification and activity forecasting utilizing any ML model. Developing a visual predictive model to assist healthcare professionals in identifying individuals with poor prognoses would be advantageous.

Consequently, a central objective of our research was to construct and evaluate ML-based survival prediction models for patients with remnant stomach cancer over a five-year period. This endeavor encompassed not only the development of multiple ML algorithms but also an emphasis on visualizing these models to gain deeper insights into their inner workings. Furthermore, our study aimed to juxtapose the efficacy of these ML models against that of traditional linear regression models, thereby shedding light on the distinctive contributions and potential superiority of ML approaches in forecasting survival probabilities for this patient population. Through visualization, we sought to enhance interpretability and transparency, enabling a comprehensive evaluation and understanding of the complex relationships learned by the ML models in the context of RGC survival prediction.

## Data and methods

### Patients

**Fig. 1:**
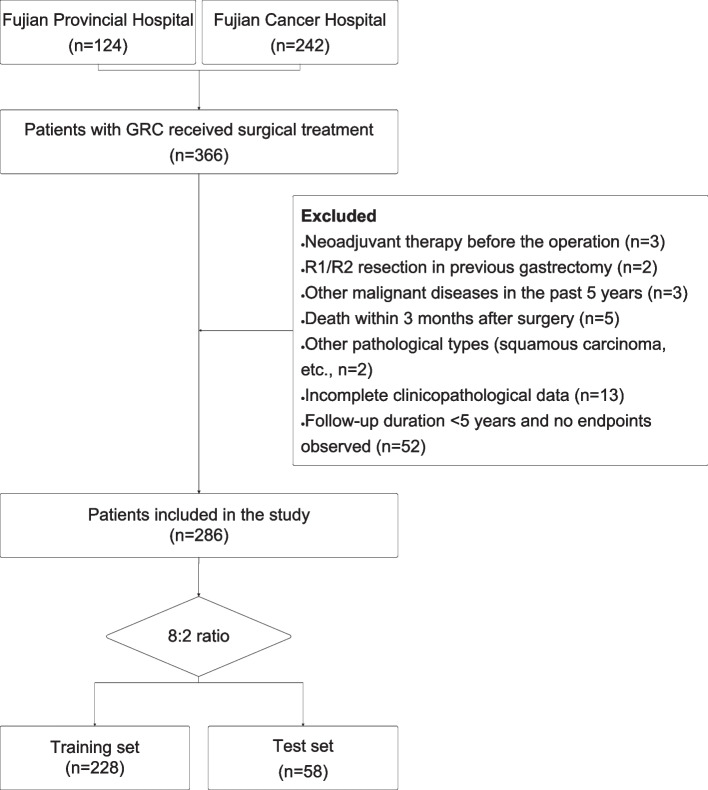
Flow diagram of the study population selected from Fujian Cancer Hospital and Fujian Provincial Hospital. According to the inclusion and exclusion criteria, a total of 286 patient were included in this study, and they were randomly cut into the training and test sets in an 8:2 ratio

Patients with RGC were enrolled at two tertiary hospitals (Fujian Provincial Hospital from June 2008 to May 2022, and Fujian Cancer Hospital from June 1999 to August 2021). RGC was characterized as an adenocarcinoma originating in the remnant stomach subsequent to a gastric resection for either a benign or malignant condition[3, 14, 42]. A total of 366 individuals participated in this study. Inclusion criteria consisted of patients who underwent surgical treatment, including radical and palliative surgery, with a follow-up duration of > 5 years or those who died. Patients with a history of neoadjuvant therapy, R1/R2 resection in previous gastrectomy, other malignant diseases within the past 5 years, death within 3 months after surgery, different pathological types, or incomplete clinicopathological data were excluded. Furthermore, patients with a follow-up duration of less than 5 years, no endpoints observed, or missing values exceeding 20% were also excluded from the study. Based on the inclusion and exclusion criteria, 286 participants remained in the study. The study’s flow chart is presented in Fig. 1. The study protocol adhered to the ethical guidelines of the 1995 Declaration of Helsinki, and was approved by the ethics committee of Fujian Cancer Hospital (ethical approval number K2021-100–01) and Fujian Provincial Hospital (ethical approval number K2022-08–034).

### Data collection

Follow-up procedures encompassed outpatient visits, hospital appointments, and telephone inquiries. The follow-up period concluded on December 31, 2023. Patients’ survival time (in months) was calculated from the date of surgery to the date of death or the end of follow-up. Retrospective analysis was conducted on preoperative information (age, initial gastric disease, initial reconstruction methods, and interval between the initial surgery and RGC resection), operative details (operative approaches, combined resections, and either curative (R0) or non-curative resections (R1/2)), and postoperative data (RGC tumor location, histopathological findings, lymph nodes ratio (LNR), venous and perineural invasions, follow-up duration, and adjuvant therapy). TNM staging was performed according to the AJCC/UICC staging criteria (8th edition) after RGC surgery[43]. Histological types were classified as highly differentiated, moderately differentiated, and lowly differentiated (including signet-ring cell carcinoma, poorly differentiated, or mucinous). Tumor locations were categorized as anastomotic and non-anastomotic sites.

### Study outcomes

The primary endpoint of the study was all-cause mortality within the 5-year follow-up period. All-cause mortality was defined as death resulting from any cause.

### Feature selection and data preprocessing

ML algorithms were implemented in Python software, and the data were organized in the format required for applying these algorithms. Samples were classified into healthy or sepsis groups based on the outcome indicators for the classification prediction model. The K-Nearest Neighbor (KNN) algorithm[44] was used to fill in missing data. To prevent non-normal distributed features from causing incorrect outcomes in ML estimators, logistic regression (with L2 penalty and c = 0.01) was employed as an external estimator, assigning weights to each feature. This approach facilitated accurate and reliable predictions in our study.

### Model development

Nine ML algorithms, including Artificial Neural Network (ANN), CatBoost, Decision Tree, Gradient Boosting Machine (GBM), Gaussian Naive Bayes (GNB), K-Nearest Neighbor (KNN), Logistic Regression, Random Forest, and Support Vector Machine (SVM), were employed to develop prognostic models. These models were compared with Linear Regression[45]. To divide the 286 patients into a training and a testing set, stratified random sampling was utilized based on the occurrence of the endpoint. The 8:2 ratio resulted in a training set of 228 patients and a test set of 58 patients.

### Model performance evaluation

Various metrics and scoring methods were employed to quantify the accuracy of predictions, including application to the evaluated estimators such as accuracy, precision, recall, and F1-score. The model’s discrimination capability was assessed using the receiver operating characteristic (ROC) curve. To prevent overfitting, repeated resampling, model fitting, and evaluation were utilized. Additionally, decision curve analysis (DCA) and calibration curves were applied to calibrate the model and provide support for probability predictions.

### Model interpretation

The Shapley Additive explanation (SHAP) package[46], a method for uniformly measuring feature importance in ML models, was employed for visualizing and explaining the prediction model. SHAP-based explanations offer a solid theoretical foundation and are the only attribution method that satisfies local accuracy, missingness, and consistency requirements[47]. The SHAP beeswarm plot provides a visual overview of the entire model, while sorting feature variables and creating scatter plots help explain the model. The SHAP dependence plot is used to visualize feature interactions and SHAP values, while the SHAP force plot enables visualization of the model at an individual level. We utilized SHAP to offer an explanation for our predictive model, which includes relevant risk factors contributing to mortality in patients with gastric stump cancer. This interpretation helps to enhance understanding of the model’s predictions and the factors influencing patient outcomes.

### Statistical analysis

Numerical variables with normal distributions were presented as mean ± SD, while those without normal distributions were represented by median (lower quartile, upper quartile). Categorical variables were expressed as the sum (percentage). Data preprocessing was performed using R software (version 3.6.3). For missing data imputation, KNN[44], Sklearn[48], and SHAP packages[46], in Python (version 3.7) were utilized respectively. The KNN package filled in missing data, while the Sklearn package built and verified the risk models. The SHAP package was used for model visualization and explanation. All models were constructed using the Sklearn package.

## Result

### Clinicopathological features of RGC

A final dataset consisting of 286 patients with RGC was obtained based on the inclusion criteria. This included 250 male patients (87.4%) and 36 female patients (12.6%). The average age of all patients was 64.3 ± 10.7 years. During a 5-year follow-up period, 142 patients (49.65%) passed away. The basic participant information is presented in Table 1. The dataset encompassed 19 clinical features, including those related to the outcome variable. To prevent later model construction from being influenced by significantly correlated features, the linear correlation between continuous numerical variables in the dataset was analyzed. As shown in Supporting Information 1, there were no significantly correlated variables (*r* < 0.8). This ensures that the constructed model is minimally affected by redundant or confounding factors.

**Table 1:** The basic information of participants

	0 (*N* = 144)	1 (*N* = 142)	Overall (*N* = 286)
Center			
Center 1	52 (36.1%)	40 (28.2%)	92 (32.2%)
Center 2	92 (63.9%)	102 (71.8%)	194 (67.8%)
Gender			
Man	127 (88.2%)	123 (86.6%)	250 (87.4%)
Woman	17 (11.8%)	19 (13.4%)	36 (12.6%)
Age			
Mean (SD)	63.7 (9.53)	64.8 (11.7)	64.3 (10.7)
Median [Min, Max]	65.0 [27.0, 86.0]	67.0 [4.00, 87.0]	66.0 [4.00, 87.0]
Interval			
Mean (SD)	20.7 (15.5)	21.6 (14.7)	21.1 (15.1)
Median [Min, Max]	20.0 [1.00, 69.0]	20.0 [1.00, 50.0]	20.0 [1.00, 69.0]
Initial gastrectomy			
Billroth I	31 (21.5%)	27 (19.0%)	58 (20.3%)
Billroth II	113 (78.5%)	115 (81.0%)	228 (79.7%)
Initial gastric disease			
Benign	85 (59.0%)	87 (61.3%)	172 (60.1%)
Malignant	59 (41.0%)	55 (38.7%)	114 (39.9%)
Location			
anastomotic	108 (75.0%)	106 (74.6%)	214 (74.8%)
non-anastomotic	36 (25.0%)	36 (25.4%)	72 (25.2%)
Grade			
High	7 (4.9%)	0 (0%)	7 (2.4%)
Inter	66 (45.8%)	48 (33.8%)	114 (39.9%)
Low	69 (47.9%)	93 (65.5%)	162 (56.6%)
Missing	2 (1.4%)	1 (0.7%)	3 (1.0%)
T stage			
Mean (SD)	2.81 (1.16)	3.61 (0.683)	3.21 (1.03)
Median [Min, Max]	3.00 [1.00, 4.00]	4.00 [1.00, 4.00]	4.00 [1.00, 4.00]
Metastasis			
No	139 (96.5%)	119 (83.8%)	258 (90.2%)
Yes	5 (3.5%)	22 (15.5%)	27 (9.4%)
Missing	0 (0%)	1 (0.7%)	1 (0.3%)
Combined resection			
No	122 (84.7%)	106 (74.6%)	228 (79.7%)
Yes	22 (15.3%)	36 (25.4%)	58 (20.3%)
Tumor size (cm)			
Mean (SD)	4.20 (2.13)	5.61 (2.61)	4.90 (2.48)
Median [Min, Max]	4.00 [0.800, 11.0]	5.00 [0.500, 15.0]	5.00 [0.500, 15.0]
Venous invasion			
No	106 (73.6%)	82 (57.7%)	188 (65.7%)
Yes	38 (26.4%)	60 (42.3%)	98 (34.3%)
Perineural invasion			
No	99 (68.8%)	62 (43.7%)	161 (56.3%)
Yes	45 (31.3%)	80 (56.3%)	125 (43.7%)
Resection margins			
No	138 (95.8%)	119 (83.8%)	257 (89.9%)
Yes	6 (4.2%)	23 (16.2%)	29 (10.1%)
Lymph nodes ratio			
Mean (SD)	0.109 (0.243)	0.365 (0.332)	0.236 (0.318)
Median [Min, Max]	0 [0, 1.00]	0.290 [0, 1.00]	0.0500 [0, 1.00]
Postoperative complications			
No	123 (85.4%)	110 (77.5%)	233 (81.5%)
Yes	21 (14.6%)	32 (22.5%)	53 (18.5%)
Adjuvant chemotherapy			
No	94 (65.3%)	89 (62.7%)	183 (64.0%)
Yes	50 (34.7%)	53 (37.3%)	103 (36.0%)

0 survivor, 1 No-survivor, Center 1 Fujian Provincial Hospital, Center 2 Fujian Cancer Hospital

### Feature variable selection

The data was prepared in the required format for implementing the ML algorithm. Nineteen observation indices were assessed for missing values. Aside from three instances where T-stage information was absent, no other variables exhibited any missing data. To fill in missing data, the K-Nearest Neighbor method was employed. For feature selection, recursive feature elimination (RFE) was utilized to enhance estimators’ accuracy scores or improve their performance on highly dimensional datasets. Logistic regression (with L2 penalty, c = 0.01, *n* = 10) was used as an external estimator to assign weights to features. This approach ensures that the selected features contribute effectively to the model’s predictive accuracy and performance.

### Model performance

**Fig. 2:**
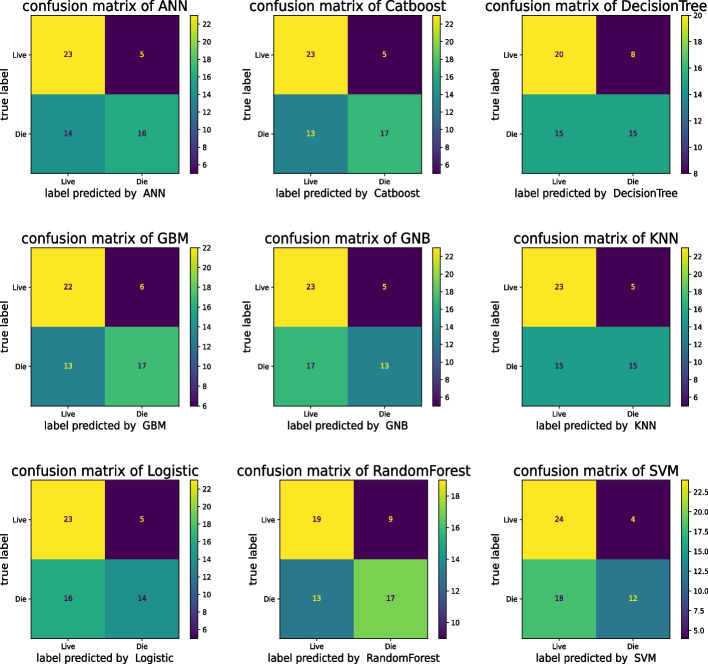
Confusion Matrices for Model Results. Numbers represent the total number of patients. The vertical axis shows the true label, and the horizontal axis shows the label predicted

**Fig. 3:**
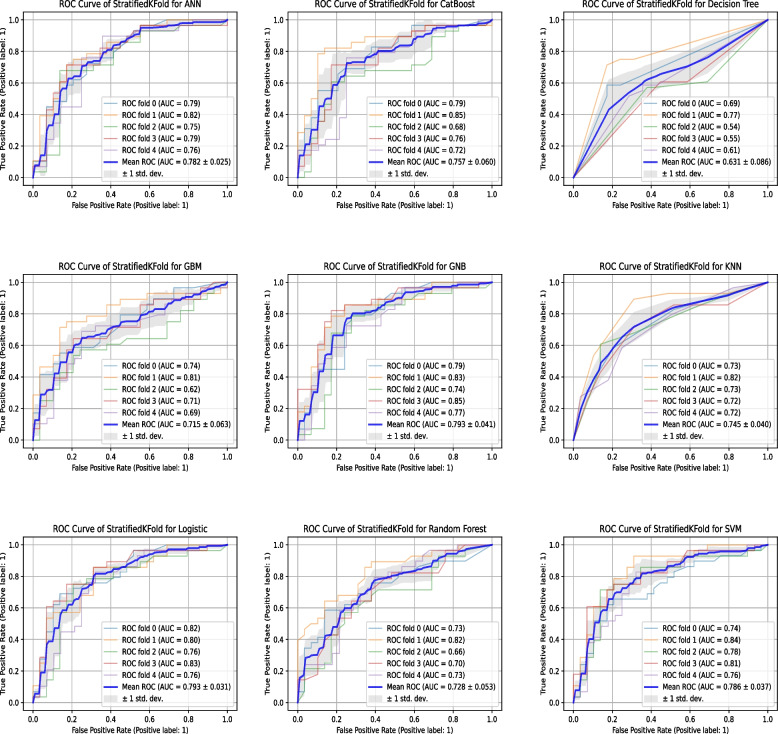
Model Evaluation. ROC Curve of Stratified K-Fold for Models

The predictive performance of the model during both training and testing, as measured by the AUC value, is detailed within Supporting Information 2. The confusion matrices illustrating the performance of the models trained on the test dataset are presented in Fig. 2. Upon comparison with conventional methodologies, the ML-built models showcased enhanced performance. Among all the models, CatBoost models emerged as having the highest f1-scores. The AUC ranged from 0.60 to 0.76 for the test set (refer to Supporting Information 3). Other metrics and scoring methods for quantifying the quality of risk models, such as False Negative Rate (FNR), False Positive Rate (FPR), False Discovery Rate (FDR), and False Omission Rate (FOR), are presented in Supplementary Table 4. Cross-validation serves as a principal method for internal validation[49], and in this study, five-fold cross-validation was employed. Table 2 showcases the performance metrics of the ML algorithms after being subjected to five-fold cross-validation on the test data. Notably, the KNN models achieved the most outstanding test set and f1-scores. Figure 3 further illuminates that, aside from the decision tree model, all other models delivered commendable performances, with an average AUC of the ROC exceeding 0.7, indicating their robustness and predictive capabilities.

**Table 2:** Metrics and Scoring for Quantifying the Quality of Model Performance with 5-Fold Stratified Cross-Validation on Test Set

	Accuracy_scores	Precision	Recall	F1-scores	AUC
	No-survivor	No-survivor	No-survivor	No-survivor	
ANN	0.67±0.09	0.66±0.07	0.65±0.07	0.67±0.09	0.782 ± 0.025
CatBoost	0.62±0.10	0.64±0.13	0.62±0.10	0.62±0.10	0.757 ± 0.060
Decision Tree	0.55±0.10	0.55±0.14	0.54±0.14	0.49±0.08	0.631 ± 0.086
GBM	0.57±0.08	0.54±0.08	0.54±0.08	0.52±0.08	0.715 ± 0.063
GNB	0.57±0.11	0.54±0.18	0.58±0.11	0.54±0.15	0.793 ± 0.041
KNN	0.74±0.09	0.75±0.10	0.74±0.09	0.74±0.10	0.745 ± 0.040
Logistic	0.66±0.09	0.66±0.08	0.66±0.09	0.65±0.09	0.793 ± 0.031
Random Forest	0.64±0.14	0.66±0.12	0.61±0.09	0.58±0.06	0.728 ± 0.053
SVM	0.69±0.09	0.70±0.09	0.69±0.09	0.69±0.09	0.786 ± 0.037

*LASSO* least absolute shrinkage and selection operator, *ANN* artificial neural network, *GBM* gradient boosting machine, *GNB* Gaussian NB, *KNN* K-nearest neighbor, *SVM* supported vector machine

**Fig. 4:**
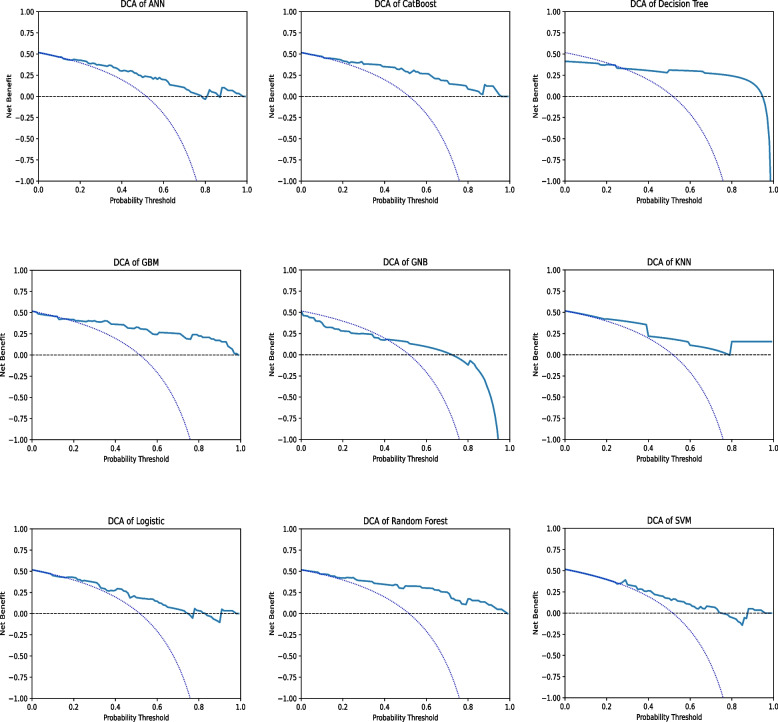
Model Evaluation. **A** Decision Curve Analysis to Evaluate the Benefits of Prediction Models. The two dashed lines reflecting the strategies of “assume all patients have the condition” (i.e., treat all) and “assume no patients have the condition” (i.e., treat none) cross at the midpoint of the preference range. The GNB model showed a significant decrease in net benefit when the threshold probabilities were greater than 80%. For the other eight models, a high net benefit was observed across a wide range of threshold probabilities

DCA is a method to determine whether using a prediction model for clinical decision-making provides benefits[50, 51]. In DCA, the net benefit is compared between two strategies: “treat all” and “treat none”. The optimal strategy is the one with the highest net benefit at a specific threshold probability. For the majority of models, the net benefit of the decision curve was higher than that for either “treat all” or “treat none” across all likely threshold probabilities. The GNB model showed a significant decrease in net benefit when threshold probabilities exceeded 80%. For the other eight models, a high net benefit was observed over a wide range of threshold probabilities. Consequently, the DCA results indicated that the constructed models could aid clinical decision-making to improve patient outcomes (Fig. 4).

**Fig. 5:**
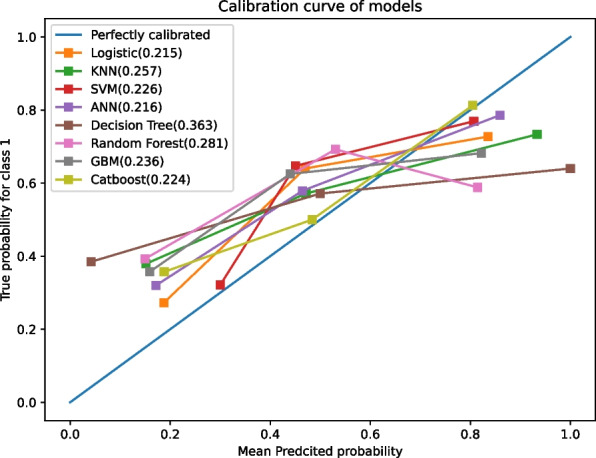
Calibration curve of models. The diagonal line serves as the reference, with which the calibration curve aligns when the predicted value matches the observed value. The curve falls below the reference when risk is overestimated and rises above it when risk is underestimated. Except for the decision tree model, the predicted values of the remaining eight models display good performance

Furthermore, the calibration curve was assessed to evaluate another measure of discrimination. The reference line is diagonal, and the calibration curve aligns with the reference when the predicted value equals the observed value. The curve is below the reference when risk is overestimated, and above when risk is underestimated. Figure 5 demonstrates that except for the decision tree model, the predicted values of the other eight models exhibited good performance.

### Visualization and explanation of models

**Fig. 6:**
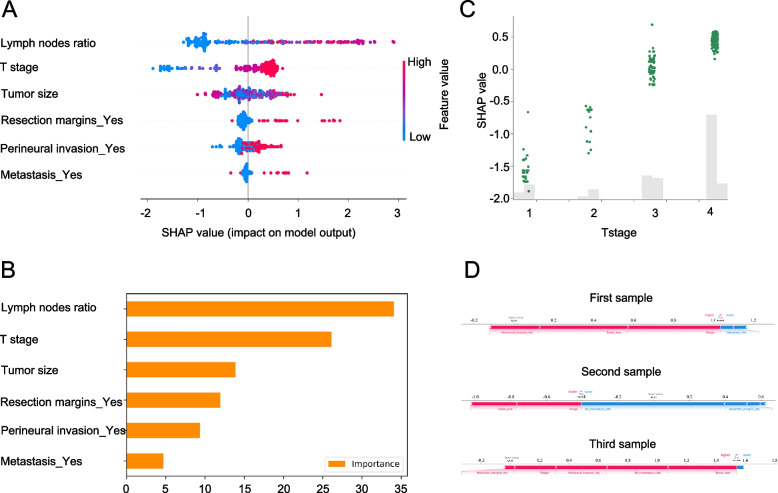
Visualization and explanation of machine learning models. **A** For the variable importance output by SHAP, the vertical axis ranks the features according to the sum of the SHAP values (the distribution of the influence of the features on the model output). **B** Variable importance ratio output using Sklearn. **C** SHAP value of the T stage. **D** Base value on the horizontal axis representing the average SHAP value of the population. The second object is relatively low-risk, with a SHAP value of -0.41. Despite the higher T stage of the individual, their low lymph nodes ratio and absence of a combined resection contribute to a decreased risk of death. The first and third objects exhibit high-risk characteristics, with SHAP values spanning from 1.0 to 2.0. Their negative factors include a high lymph nodes ratio, advanced T stage, large tumor size, perineural invasion, and a combined resection

The 5-year death prediction model based on ML techniques performed satisfactorily in terms of model validity and clinical net benefit. Nonetheless, the opaque nature of ML models creates a lack of transparency. SHAP values reveal the individual contributions of each feature to the final prediction, effectively clarifying and interpreting model predictions for specific patients. After sorting features, SHAP was applied to distinguish the feature values for the selected variable (Fig. 6A). To explain the CatBoost-based model, the SHAP summary plot was utilized. The study findings suggested that a high lymph node ratio (red) had a negative impact on prognosis, while a low lymph node ratio (blue) contributed positively. Concurrently, a high Tstage (red) showed a negative effect on prognosis, whereas a low Tstage (blue) had a positive influence on the patient’s outlook. The results corresponded to those concerning resection margins, positive metastasis, and perineural invasion.

After several years of development, traditional ML methods have become capable of displaying feature variables. However, these methods fail to demonstrate the positive and negative relationships between features within the model (Fig. 6B).

The SHAP Dependence Plot enables visualization of the effects within the model. Each dot represents a sample (Fig. 6C). It was observed that as the T stage increased, so did the SHAP values. The SHAP Force Plot illustrates the individual level within the model. Figure 6D demonstrates the significance of influencing factors for the three subjects in the RGC. In comparison to the first sample (SHAP, 1.03) and third sample (SHAP, 1.54), the second sample (SHAP, -0.41) belonged to the low-risk group, possessing a decreased risk of 5-year death. Variables influencing the model’s outcomes are listed below the horizontal axis. Different individuals might have identical or slightly varying key variables affecting their outcomes.

## Discussion

Our research harnessed ML techniques to create a set of ML models skilled at forecasting five-year survival prognoses for RGC following surgery. This is the first investigation to examine prognostic risk factors for RGC utilizing ML models. Through the development and validation of this model, we have showcased its consistent performance and superior reproducibility. Significantly, our risk model not only demonstrates robust stability compared to conventional techniques but also addresses the ‘black box’ issue associated with ML models by incorporating model visualization techniques. By visualizing the model, we enable healthcare professionals to more effectively discern post-surgery survival outcomes. These predictive indicators potentially grant clinicians an enhanced ability to tailor care strategies, thereby optimizing risk factor management for high-risk patients.

The proficiency, user-friendliness, and resilience of ML models in recognizing complex data significantly surpass traditional statistical models, overcoming their limitations regarding statistical efficiency[49]. In ML models, classes can be utilized for feature selection or dimensionality reduction to enhance the model’s accuracy score or improve its performance on high-dimensional datasets[52]. Gradient boosted decision trees (GBDTs), including XGBoost, LightGBM, and CatBoost, are potent tools for big data classification tasks. Our method provides not only a precise and clinically feasible technique for predicting RGC patient survival outcomes but also enhances the interpretability of the predictions. The SHAP value quantifies each feature marker’s contribution to the model’s identification results, enabling comprehensive global explanations[46, 53, 54]. The predictive capacity of a clinical factor in the XGBoost model elevates as the average absolute SHAP value of each factor rises. To obtain a uniform perspective, these factors were consolidated, and SHAP interpretation drew from individual patients. SHAP effectively addresses multicollinearity issues and determines whether an influence is beneficial, thanks to its ability to consider both individual factor effects and their synergies[41]. According to the SHAP values, LNR, T stage, tumor size, resection margins, perineural invasion, and distant metastasis were determined as the most crucial factors in identifying five-year survival prognoses for RGC. In essence, these factors can be considered an optimal subset representing the key players in survival risk assessment for RGC patients. The interpretability of the optimal subset stems from capturing and visualizing the effect direction of each feature and its contribution size to the prediction. This enables clinicians to gain specific insights into how individual predictions are influenced by various variables, affording a personalized, fine-grained understanding of different patients’ prognoses.

Most reports indicate that RGC is often diagnosed at an advanced stage, leading to a relatively low rate of curative resection and unfavorable prognosis. This suggests that RGC may possess distinct biological characteristics from primary GC[1, 55, 56]. However, some researchers have compared RGC to primary GC and found no significant difference in survival rates between the two[57–59]. A few studies have investigated the clinicopathologic features and prognosis of RGC, but consensus has not been reached yet[1, 60, 61]. Similar to prior research[56, 62, 63], our study noted that more than 80% of RGC patients were male. This may be attributed to the fact that men are more susceptible to developing both gastroduodenal ulcers and GC[64, 65].

In the majority of studies, RGC lymph node staging adheres to the UICC/AJCC grading criteria. However, in first-time GC patients, postoperative lymph node drainage changes and the lymph nodes detected by RGC cannot comprehensively determine the N stage, particularly given the occurrence of RGC after GC. The total number of postoperative lymph node dissections during re-surgery typically does not exceed 10, which is significantly fewer than the number of lymph nodes dissected by RGC after surgery for benign lesions. This may lead to inaccurate staging. A study analyzed the prognostic significance of LNR in resectable RGC using retrospective propensity score matching and found that LNR served as an independent prognostic factor for RGC, while the number of positive lymph nodes did not act as an independent prognostic factor[42]. Our study reinforced this notion using an ML method. Therefore, LNR may be a more dependable prognostic factor for RGC patients. However, some studies suggest that LNR is not superior to the number of positive lymph nodes[66]. Further analysis incorporating data from multiple centers with larger sample sizes is necessary.

Another study identified lymphatic invasion and pathological T stage as risk factors for lymph node metastasis in RGC[67]. Many researchers have proposed that high rates of adjacent organ invasion and lymph node metastasis contribute to RGC’s poorer prognosis[19, 20]. Nonetheless, one study found pathological T stage and venous invasion to be significant independent risk factors for survival among RGC patients[68]; however, pathological N stage showed no significant association with long-term survival[68]. This contradicts our study’s findings. In our research, venous infiltration was not included in the prognostic model, suggesting it is not an independent prognostic factor, and nerve invasion plays a crucial role. Given their small sample size (65 cases) and single-center retrospective study, the prognostic value of venous infiltration deserves further examination. It has been demonstrated that tumor site affects RGC’s prognosis[22, 23, 27]. RGC’s tumor location is a vital factor for predicting recurrence patterns and overall survival[69]. However, in our study, tumor location at the anastomotic site did not act as an independent prognostic factor, which aligns with previous reports[70, 71].

The current study unavoidably has several limitations. Firstly, due to its retrospective nature, there was selection bias. Secondly, the sample size was relatively small. Thirdly, some crucial information was incomplete or missing, likely caused by difficulties in gathering data about the initial operation. Further prospective studies involving RGC patients are necessary to comprehensively explore the clinicopathological characteristics of RGC.

Given the primary aim of our research to optimize the use of pathological features in predicting mortality risks for post-gastrectomy GC patients, we intentionally confined our analysis to these specific characteristics. Consequently, we did not incorporate other potentially influential mortality risk factors, such as comorbidities, laboratory indices, and other clinical attributes for stratification purposes. This deliberate focus on pathology data alone may have limited the model's ability to achieve its maximum predictive capacity. Nonetheless, this study serves as a foundational step towards refining risk prediction. Moving forward, we plan to extend our work by integrating additional clinical indicators and biomarkers to construct a more refined and comprehensive predictive model. Such a holistic approach will likely enhance the precision and practicality of risk assessment in this patient population.

## Conclusion

In summary, utilizing the CatBoost ML model to develop a prognostic risk model for RGC can effectively assist clinicians in predicting patient outcomes, outperforming traditional ML methods. Moreover, combining SHAP and ML may serve as a suitable approach to identify individuals with poor prognoses.

### Supplementary Information

**Additional file 1:** **Supporting Information 1.** Correlation Matrix of different variables.

**Additional file 2:** **Supporting Information 2.** Model Evaluation. ROC Curves for Test and Training Sets.

**Additional file 3:** **Supporting Table 3.** Metrics and scoring for quantifying the Performance Quality of Risk Models on Test Set

**Additional file 4:** **Supporting Table 4.** Other metrics and scoring for quantifying the quality of risk models

## Data Availability

For reproducibility and transparency, the original data and the code used in this study are made publicly available at: https://github.com/Xiaofan2023/FJMUZZW-ML-Diagnostic-Model.git.

## Abbreviations

ML: Machine learning
RGC: Remnant gastric cancer
GC: Gastric cancer
AUC: Area under the curve
DCA: Decision curve analysis
SHAP: SHapley Additive exPlanations
LNR: Lymph nodes ratio
PUD: Peptic ulcer disease
AJCC: American Joint Committee on Cancer
UICC: Union for International Cancer Control
KNN: K-nearest neighbor
ANN: Artificial neural network
GBM: Gradient boosting machine
GNB: Gaussian NB
SVM: Support vector machine
ROC: Receiver operating characteristic
DCA: Decision curve analysis
RFE: Recursive feature elimination
FNR: False Negative Rate
FDR: False Discovery Rate
FOR: False Omission Rate
GBDT: Gradient boosted decision tree
