# Supplementary figures and images for: Identification of prognostic signatures in remnant gastric cancer through an interpretable risk model based on machine learning: a multicenter cohort study
Source: BMC Cancer. 2024 Apr 30;24:547. doi: 10.1186/s12885-024-12303-9 (PMC11062017; doi:10.1186/s12885-024-12303-9)

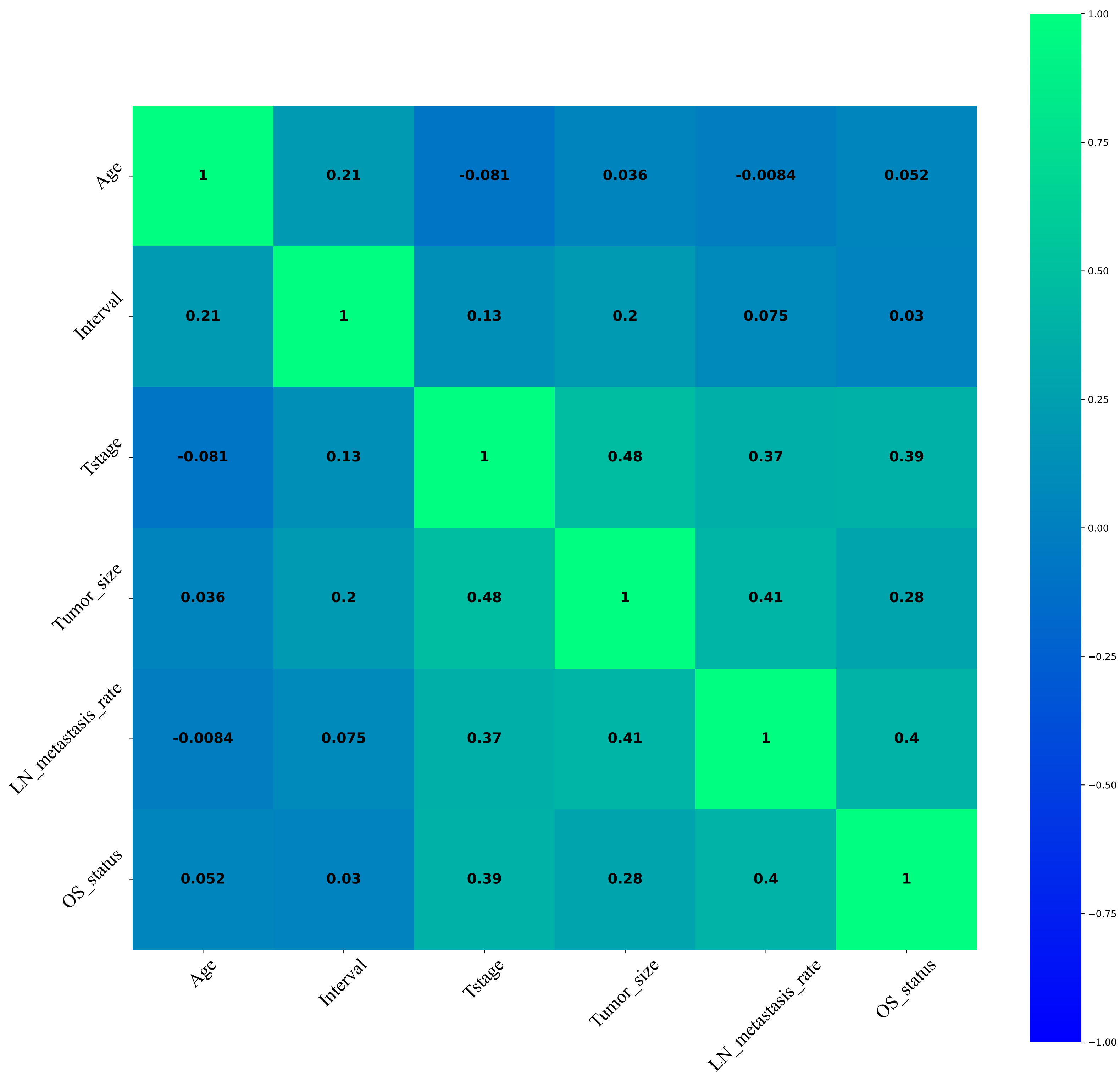

Supplement: Supplementary file 1 — Additional file 1: Supporting Information 1. Correlation Matrix of different variables. [file 12885_2024_12303_MOESM1_ESM.pdf]
