# Supplementary material for: Identification of prognostic signatures in remnant gastric cancer through an interpretable risk model based on machine learning: a multicenter cohort study
Source: BMC Cancer. 2024 Apr 30;24:547. doi: 10.1186/s12885-024-12303-9 (PMC11062017; doi:10.1186/s12885-024-12303-9)
Supplement: Supplementary file 2 — Additional file 2: Supporting Information 2. Model Evaluation. ROC Curves for Test and Training Sets. [file 12885_2024_12303_MOESM2_ESM.pdf]

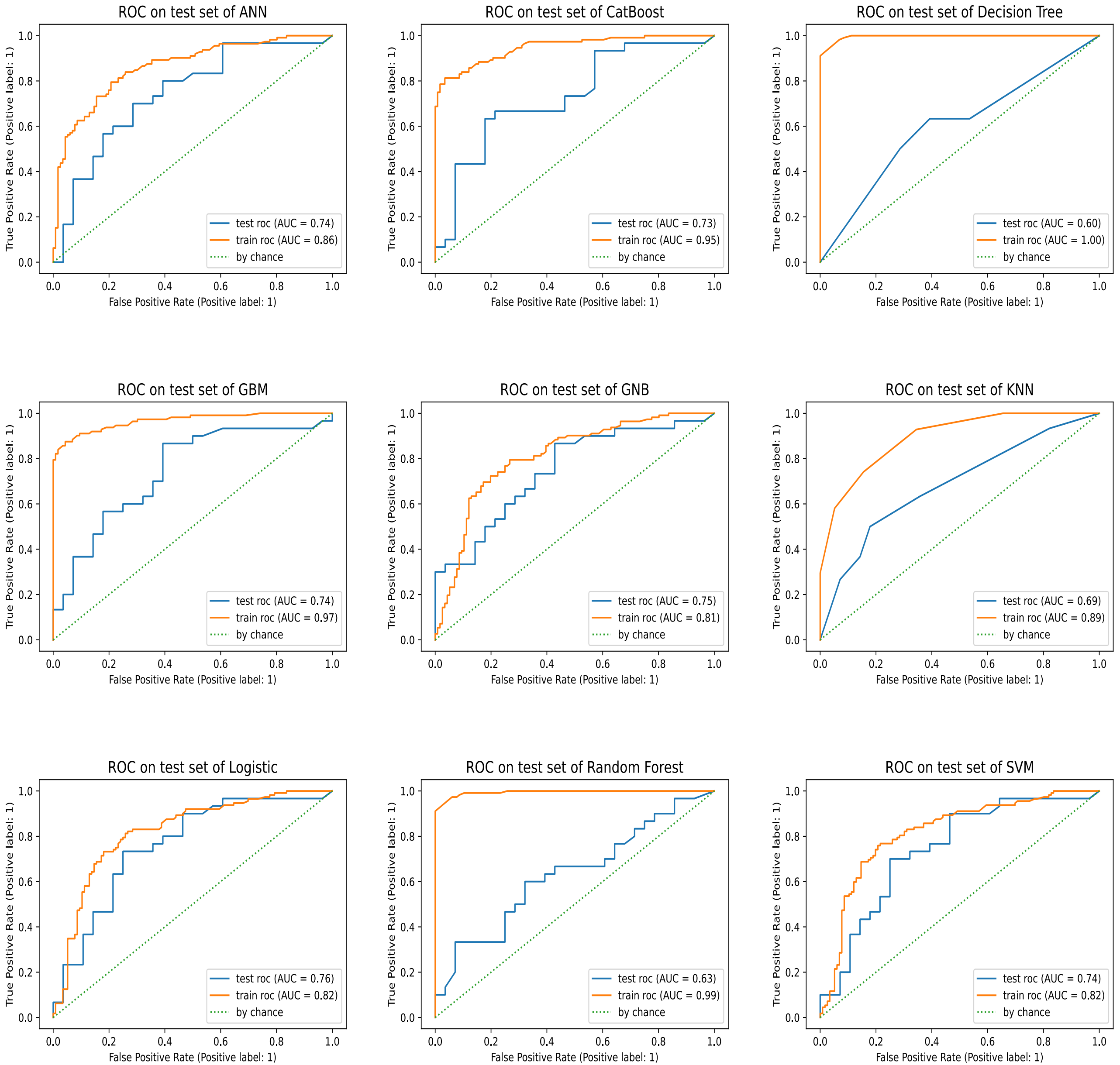
ROC on test set of ANN
test roc (AUC = 0.74)
train roc (AUC = 0.86)
by chance
ROC on test set of CatBoost
test roc (AUC = 0.73)
train roc (AUC = 0.95)
by chance
ROC on test set of Decision Tree
test roc (AUC = 0.60)
train roc (AUC = 1.00)
by chance
ROC on test set of GBM
test roc (AUC = 0.74)
train roc (AUC = 0.97)
by chance
ROC on test set of GNB
test roc (AUC = 0.75)
train roc (AUC = 0.81)
by chance
ROC on test set of KNN
test roc (AUC = 0.69)
train roc (AUC = 0.89)
by chance
ROC on test set of Logistic
test roc (AUC = 0.76)
train roc (AUC = 0.82)
by chance
ROC on test set of Random Forest
test roc (AUC = 0.63)
train roc (AUC = 0.99)
by chance
ROC on test set of SVM
test roc (AUC = 0.74)
train roc (AUC = 0.82)
by chance
True Positive Rate (Positive label: 1)
False Positive Rate (Positive label: 1)
0.0
0.2
0.4
0.6
0.8
1.0
