# Supplementary material for: Identification of prognostic signatures in remnant gastric cancer through an interpretable risk model based on machine learning: a multicenter cohort study
Source: BMC Cancer. 2024 Apr 30;24:547. doi: 10.1186/s12885-024-12303-9 (PMC11062017; doi:10.1186/s12885-024-12303-9)
Supplement: Supplementary file 3 — Additional file 3: Supporting Table 3. Metrics and scoring for quantifying the Performance Quality of Risk Models on Test Set [file 12885_2024_12303_MOESM3_ESM.docx]

Table 3 Metrics and Scoring for quantifying the Performance Quality of Risk Models on Test Set

|  | Accuracy_scores | Precision | Recall | F1-scores | AUC |
| --- | --- | --- | --- | --- | --- |
| Linear | 0.14 |  |  |  |  |
| LASSO Linear | 0.13 |  |  |  |  |
| ANN | 0.67 | 0.76 | 0.53 | 0.63 | 0.74 |
| CatBoost | 0.69 | 0.77 | 0.57 | 0.65 | 0.73 |
| Decision Tree | 0.60 | 0.65 | 0.50 | 0.57 | 0.60 |
| GBM | 0.67 | 0.74 | 0.57 | 0.64 | 0.74 |
| GNB | 0.62 | 0.72 | 0.43 | 0.54 | 0.75 |
| KNN | 0.66 | 0.75 | 0.50 | 0.60 | 0.69 |
| Logistic | 0.64 | 0.74 | 0.47 | 0.57 | 0.76 |
| Random Forest | 0.62 | 0.65 | 0.57 | 0.61 | 0.63 |
| SVM | 0.62 | 0.75 | 0.40 | 0.52 | 0.74 |

LASSO, least absolute shrinkage and selection operator; ANN, artificial neural network; GBM, gradient boosting machine; GNB, Gaussian NB; KNN K-nearest neighbor; SVM, supported vector machine.
