# Supplementary material for: Identification of prognostic signatures in remnant gastric cancer through an interpretable risk model based on machine learning: a multicenter cohort study
Source: BMC Cancer. 2024 Apr 30;24:547. doi: 10.1186/s12885-024-12303-9 (PMC11062017; doi:10.1186/s12885-024-12303-9)
Supplement: Supplementary file 4 — Additional file 4: Supporting Table 4. Other metrics and scoring for quantifying the quality of risk models [file 12885_2024_12303_MOESM4_ESM.docx]

Table 3 Other metrics and scoring for quantifying the quality of risk models

|  | TPR | FNR | FPR | TNR | FDR | FOR |
| --- | --- | --- | --- | --- | --- | --- |
| ANN | 0.72 | 0.67 | 0.62 | 0.76 | 0.82 | 0.53 |
| CatBoost | 0.78 | 0.69 | 0.64 | 0.77 | 0.82 | 0.57 |
| Decision Tree | 0.79 | 0.60 | 0.57 | 0.65 | 0.71 | 0.50 |
| GBM | 0.81 | 0.67 | 0.63 | 0.74 | 0.79 | 0.57 |
| GNB | 0.63 | 0.62 | 0.57 | 0.72 | 0.82 | 0.43 |
| KNN | 0.67 | 0.66 | 0.61 | 0.75 | 0.82 | 0.50 |
| Logistic | 0.67 | 0.64 | 0.59 | 0.74 | 0.82 | 0.47 |
| Random Forest | 0.79 | 0.62 | 0.59 | 0.65 | 0.68 | 0.57 |
| SVM | 0.66 | 0.62 | 0.57 | 0.75 | 0.86 | 0.40 |

TPR, True Positive Rate; FNR, False Negative Rate; FPR, False Positive Rate; TNR, True Negative Rate; FDR, False Discovery Rate; FOR, False Omission Rate.
